# Projection Stereolithography 3D-Printed Bio-Polymer with Thermal Assistance

**DOI:** 10.3390/polym15224402

**Published:** 2023-11-14

**Authors:** Hao Pu, Yuhao Guo, Zhicheng Cheng, Zhuoxi Chen, Jing Xiong, Xiaoyang Zhu, Jigang Huang

**Affiliations:** 1 Department of Mechanical Engineering, Sichuan University, Chengdu 610065, China; 2 Advance Research Institute, Chengdu University, Chengdu 610106, China; 3 Shandong Engineering Research Center for Additive Manufacturing, Qingdao University of Technology, Qingdao 266520, China

**Keywords:** 3D printing, stereolithography, solute loading, HEMA

## Abstract

A stereolithography process with thermal assistance is proposed in this work to address the tradeoff between the flowability and the high concentration of solute loadings at room temperature, through which the improved performance of polymers prepared using stereolithography 3D printing can be achieved. For the experiment, polyethylene glycol diacrylate (PEGDA) with a high molecular weight of 4000 is adopted to improve the mechanical properties of 2-Hydroxyethyl methacrylate (HEMA). For the polymer of HEMA, the highest soluble concentration of PEGDA is about 20 wt% at 25 °C (room temperature) while the concentration could be raised up to 40 wt% as the temperature increases to 60 °C. The 3D printing tests showed that the objects could be easily fabricated with the HEMA polymer loaded with 40 wt% of PEGDA through the thermally assisted projection stereolithography technology. By adding the 40 wt% of PEGDA, the Young’s modulus has been enhanced by nearly 390% compared to the HEMA resin without solute, of which the Young’s modulus is 63.31 ± 2.72 MPa. The results of the cell proliferation test proved that the HEMA resin loaded with PEGDA led to a better biocompatibility compared to the HEMA resin without the loading of the PEGDA solute. All of the results demonstrate that the polymer loaded with high solute is feasible to be precisely 3D-printed using the projection stereolithography process with thermal assistance, and the improved mechanical properties are beneficial for biomedical applications.

## 1. Introduction

Projection stereolithography 3D printing produces each layer by projecting the pattern onto the surface of photosensitive resins selectively based on photopolymerization [1]. Projection stereolithography 3D printing technology has developed tremendously in recent years, which has meant that finely fabricated complex three-dimensional structures have been able to be created at high resolution and with high efficiency [2,3,4]. The digital micromirror device (DMD) was introduced to the projection stereolithography process as a dynamic mask by Sun et al. in 2005 [5] to realize 3D printing with sub-micrometer resolution. To improve the fabrication speed as well as the surface quality, Tumbleston et al. [6,7] first proposed a projection stereolithography 3D printing technology known as the continuous liquid interface production (CLIP). Recently, conformal stereolithography [8], multi-material 3D printing [9], the HARP (high-area rapid printing) process [10], volumetric 3D printing [11,12], and nano 3D printing with two-step absorption [13,14] have been proposed, which has significantly improved the manufacturing capability of projection stereolithography 3D printing.

Meanwhile, progress has been made in printable materials for stereolithography. To seek the high-level functionalities and properties of printed objects, kinds of physical fields were used during the in situ 3D printing [15]. Chen et al. presented electrically assisted 3D printing to fabricate hierarchical structures with aligned graphene nanoplatelets [16]. In the acoustic field, the particles in the photopolymer show an ordered arrangement, whose density can be controlled by the acoustic field. In this way, the ordered multi-scale microstructures can be fabricated from the functional composited materials [17,18,19]. Zhai et al. introduced ultrasound-assisted digital light 3D printing to achieve directed self-assembly manufacturing for bioengineering applications [20]. Similarly, the parts with programmed particle orientation are able to be 3D-printed with magnetic assistance [21,22]. For stereolithography-based 3D printing, the polymers with high solute loadings or high viscosity are challenged to be printed at room temperature due to low flowability. However, 3D printing with thermal assistance is expected to handle polymers with low flowability, which always possess excellent mechanical properties [23,24]. For example, Sangermano et al. showed that the epoxy resin with high viscosity could be precisely 3D-printed by using thermally assisted stereolithography technology, and printed structures with good thermomechanical properties were obtained [25].

In general, the performance of photopolymer resins is related to the choice of additives, which serve to enhance the performance of the photopolymer resins [26,27,28]. The performance and functionality of photopolymer resins are still linked to additives, and the performance and functionality of the photopolymer resins relate positively to the concentration of additives [29]. Our previous [30] work proved that the photopolymer with high solute loading could be 3D-printed with thermal assistance and the mechanical properties of the polymer were dramatically improved. However, the previous work was limited to materials with similar molecular structures (PEGDA and PEG were chosen to be the solute and matrix resin). To expand the available materials for thermally assisted projection stereolithography 3D printing, in this work, the printability of a biopolymer of 2-Hydroxyethyl methacrylate (HEMA) was investigated with high solute loading at a high temperature of 60 °C, while polyethylene glycol diacrylate (PEGDA), which is commonly used in stereolithography 3D printing, was used as a solute. The mechanical properties and biocompatibility of HEMA resins were investigated separately by performing compression and tensile tests and cell proliferation experiments on HEMA resins with and without solute addition. This work is expected to present a preliminary idea and method for future research on projection stereolithography with the thermally assisted 3D printing of the polymer with a low flowability.

## 2. Experimental Section

### 2.1. Projection Stereolithography 3D Printing System

**Figure 1:**
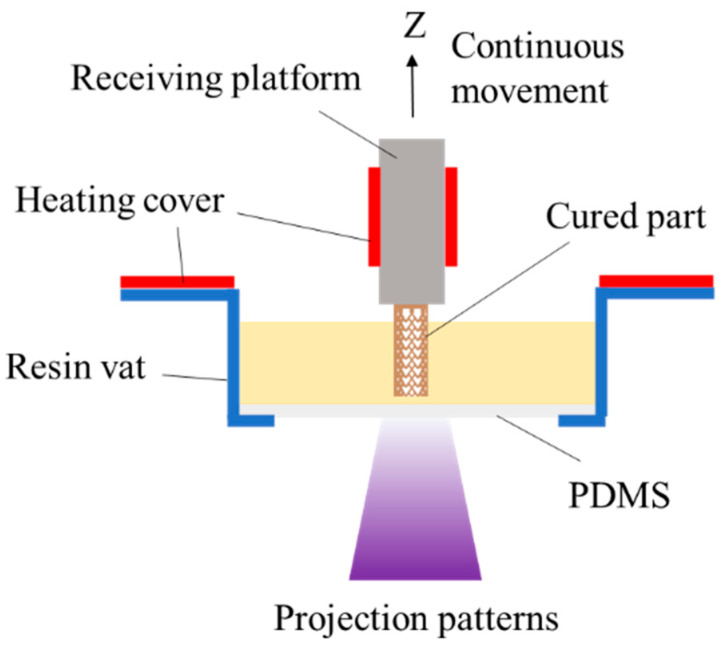
The projection stereolithography 3D printing with thermal assistance.

A digital light processing (DLP) projector (Fuzhou Gyinda Photoelectric Technology Co., Ltd., Fuzhou, China), which possesses a 405 nm light source, was employed to projected thermal assistance stereolithography 3D printing systems. To improve the fabrication resolution, the single pixel size was zoomed to 5 × 5 μm^2^ using an optical lens (Universe Kogaku America, Inc., Long Island, NY, USA). As shown in Figure 1, the cured part was fabricated in a resin vat and adhered to the printing receiving platform, and the heating covers were, respectively, added to the superficies of the resin vat and the printing receiving platform, which control the temperature of the system during 3D printing. By projecting patterns onto the photosensitive resin layer by layer, the designed structure could be printed as the receiving platform moved along the Z+ direction. The Projection Stereolithography 3D Printing System utilizes a polydimethylsiloxane (PDMS) membrane as the oxygen-permeable window, which creates an oxygen-containing dead zone below the projection plane. The oxygen-containing dead zone is able to inhibit photopolymerization between the oxygen-permeable window and the cured part, which allows the cured part to be exposed continuously without stopping from layer to layer as it is printed. Then, the fabrication output could be significantly improved while the staircase effect was suppressed to achieve the high surface quality [6].

### 2.2. Material Preparation

The materials used in this study are formulated from commercially available photopolymers, photoinitiator, and UV absorber. HEMA (CAS: 868-77-9) was used as a photopolymer and ethylene glycol dimethacrylate (EGDMA, CAS: 97-90-5) was dissolved in HEMA with the concentration of 5 wt% to improve the printability. The polyethylene glycol diacrylate (PEGDA-4000, CAS: 26570-48-9) of molecular weight 4000, which had been ultrasonically oscillated for 30 min at room temperature, was added to the polymer in the concentration range of 10 wt%, 20 wt%, 30 wt%, and 40 wt%. Each mixed solution was treated with ultrasonic oscillation for 30 min at the temperature of 60 °C. The HEMA/EGDMA solution without PEGDA was set as the control group. The photoinitiator of ethyl (2,4,6-trimethylbenzoyl) phenylphosphinate (TPO-L, CAS: 84434-11-7) and the UV absorber of Sudan I (CAS: 842-07-9) were dissolved into each designed solution with the concentrations of 0.5 wt% and 0.05 wt%, respectively. Then, the designed resins were ready for 3D printing.

### 2.3. Biocompatibility Experiment

The cell proliferation was conducted for the biocompatibility study. The cells used in this study were the mouse osteoblast cell line MT3T3-E1 purchased from American Type Culture Collection (ATCC, Rockville, MD, USA). The procedure for the cell proliferation experiments was specified as follows: the printed three-dimensional structures were first immersed in ethanol for 48 h, and after being removed from the ethanol were rinsed several times, which was performed to remove Sudan I. Then, the purchased mouse osteoblast cell line was seeded onto the three-dimensional structure from which Sudan I had been removed. The seeded completed 3D structures were cultured in α-MEM containing 10% FBS and 1% antibiotics (100 U/mL for both penicillin and streptomycin) at 37 °C and 5% CO_2_. To assess cell viability, we used the MTT assay. Specifically, the treated cells were incubated in a 10% solution of 3-(4,5-dimethylthiazol-2-yl)-2,5- diphenyltetrazolium bromide (MTT) for 4 h at 37 °C, and the supernatants were removed. Then, the formazan crystals were dissolved in 150 μL DMSO, and their light absorption value can be measured by using a specific wavelength, which can reflect the number of live cells.

### 2.4. Characterization

The scanning electron microscopy (SEM) of Hitachi S4800 (Tokyo, Japan) was used to study the morphology and microstructure of printed models. Microcomputer electronic universal material testing machine (Group Lung Instrument Co., Ltd., Xiamen, China) was used to test the mechanical properties of printed three-dimensional structures. The objects printed using designed resins were analyzed with an X-ray diffractometer (XRD, Empyrean, PANalytical B.V., Malvern, UK) and an infrared spectrometer (INVENIO R, Bruker, Germany). To detect the cell viability, a cck-8 kit was used and the result was recorded with a microplate reader (Bio-Rad, Foster, CA, USA) by using the wavelength of 490 nm.

## 3. Results and Discussion

### 3.1. 3D Printing with Thermal Assistance

**Figure 2:**
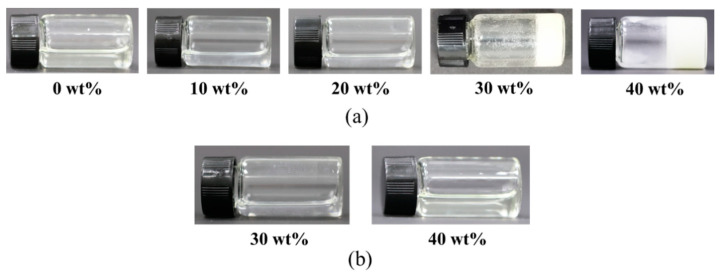
The HEMA resins. (**a**) Resins at room temperature. (**b**) Resins at the high temperature of 60 °C.

As shown in Figure 2a, PEGDA-4000 can be dissolved in the HEMA solution with the concentration of 20 wt% while PEGDA-4000 crystallizes as the concentration increases to 30 wt% at room temperature. The results show that the high solute loading of PEGDA-4000 could not be fully dissolved in the HEMA resin at room temperature, which meant it could not be used for stereolithography 3D printing After a few attempts, the high solute loading of PEGDA-4000 was completely dissolved in HEMA solution at 60 °C, which can be seen in Figure 2b, indicating the enhanced solubility at the high temperature.

The curing depth experiment is conducted for each resin, which aims to validate the printability of the HEMA resins. Specifically, for each dosage, five suspended beams were 3D-printed, and the thicknesses of which were measured. Then, the curing depth was the average of the five measurements. The curing depth, the thickness of the UV-polymerized layer, is determined by the light energy density (dose) within the exposure area. According to the exponential law of light absorption within the resin, the light intensity *I*(*z*) via the penetrating thickness z equals [31]:

*I*(*z*) = *I*_0_exp(−*z*/*D_p_*) (1)

in which *I*_0_ is the intensity at the surface, and *D_p_* is the maximum depth that light can penetrate the resin. When the light intensity decreases to the value below the critical dose, the polymerization of the resin stops. The curing depth *C_d_* can thus be calculated with the equation:

*C_d_* = *D_p_*ln(*E*_0_/*E*_c_) (2)

where *E*_0_ = *I*_0_*t* is the dose at the top surface of the liquid resin, and *E*_c_ is the critical dose of photopolymerization.

**Figure 3:**
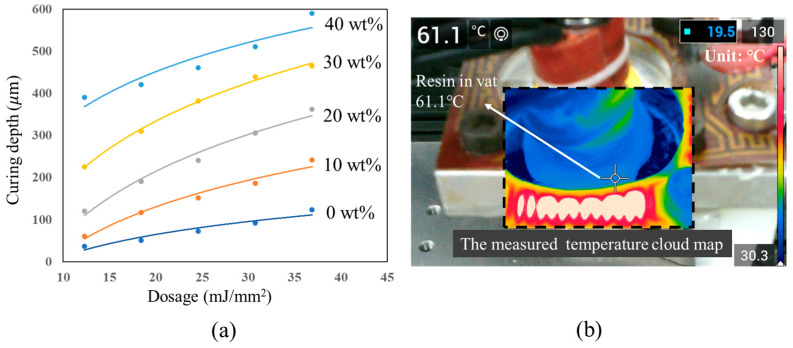
3D printing with thermal assistance. (**a**) The curing depth test, and (**b**) the real-time temperature of the resin during printing.

The HEMA resins with the solute PEGDA-4000 concentrations below 20 wt% are 3D-printed at room temperature while the resins with the solute concentrations of 30 wt% and 40 wt%, because of crystallization, are printed at the temperature of 60 °C. The curing depth curves of the designed resins with different concentrations at different temperatures, respectively, are shown in Figure 3a, which indicates that the photopolymerization of the HEMA resins is controllable for thermal assistance 3D printing. The resin containing solute PEGDA-4000 showed more depth of cure compared to the HEMA resin without solute at the same UV energy dosage. Meanwhile, the curing depth becomes large with the increase in the PEGDA-4000 concentration, implying the polymerization process is promoted by adding the PEGDA-4000 solute. The temperature distribution in and around the resin vat during thermal assistance 3D printing is shown in Figure 3b. The temperature distribution is represented by colors in the cross target while the color bar shows the corresponding connection, which indicates that the printing process is carried out at 61.1 °C.

### 3.2. 3D Printing Experiments

**Figure 4:**
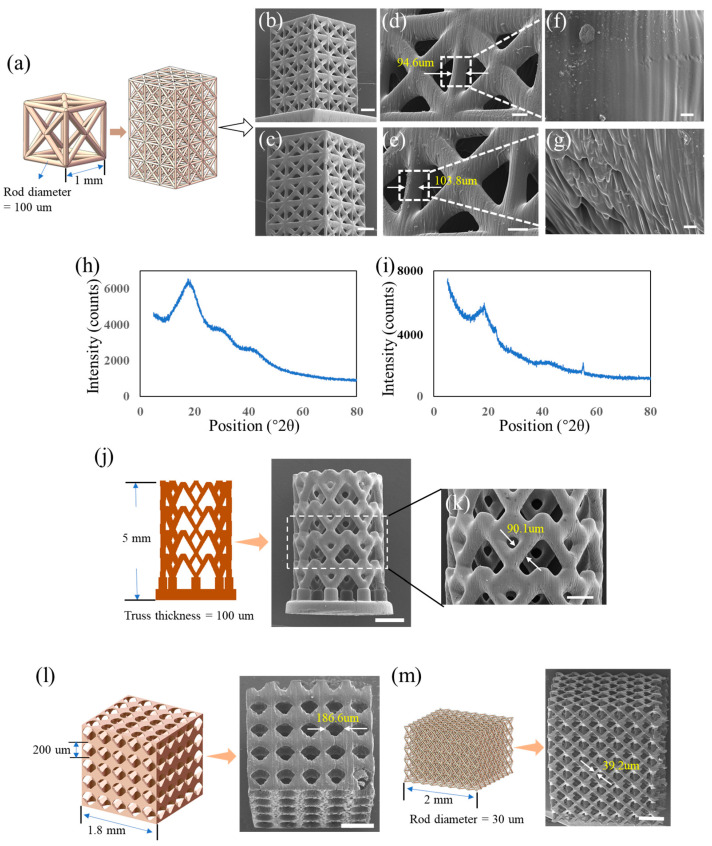
Thermal assistance 3D printing experiment. (**a**) The lattice model, (**b**) the part fabricated with the HEMA resin without PEGDA-4000 solute, (**c**) the part fabricated with the HEMA resin with 40 wt% of PEGDA-4000 solute; (**d**,**e**) are the magnified images for (**b**,**c**), respectively; (**f**,**g**) are the surface images for selected areas shown in (**d**,**e**), respectively; (**h**,**i**) are the results of infrared spectra analysis for the HEMA resin without and with PEGDA-4000, respectively; (**j**,**k**) are the images of the stent model fabricated with the HEMA resin with 40 wt% of PEGDA-4000 solute; (**l**,**m**) are the models with microfeatures fabricated with the HEMA resin with 40 wt% of PEGDA-4000 solute. Scale bar: (**b**,**c**): 1 mm, (**d**,**e**): 100 μm, (**f**,**g**): 20 μm, (**j**): 500 μm, (**k**): 200 μm, (**l**,**m**): 500 μm.

The experiments in this work are implemented to study the performance of designed high-solute-loading resins on 3D printing microstructures with thermal assistance. For each resin, five samples were 3D-printed for each structure and the sample used for the characterization was chosen randomly. To study the co-polymerization of HEMA and PEGDA, the HEMA resin with the higher concentration of PEGDA is the priority candidate. When the co-polymerization of resin with the higher concentration is verified to be feasible, the resins with lower concentrations are supposed to be 3D printable. Thus, for the 3D printing experiment, the HEMA resin with 40 wt% PEGDA-4000 loading was chosen to compare to the HEMA rein without loading. The model shown in Figure 4a was printed with the two resins. From the results, both the HEMA resin without solute printed at room temperature (Figure 4b,d) and the HEMA resin with 40 wt% PEGDA-4000 loading printed at the temperature of 60 °C (Figure 4c,e) show excellent performance on 3D printing microstructures. Meanwhile, the surface for each 3D-printed part was characterized. The result indicated that the surface of the part printed with HEMA resin without solute (Figure 4f) was much smoother than that of the part printed with HEMA resin with the high concentration loading of PEGDA-4000 (Figure 4g). The is probably because the solute PEGDA-4000 crystallizes during the 3D printing process, XRD analyses are, respectively, performed on samples made from HEMA resin with and without PEGDA-4000 solute.

The XRD pattern for the sample fabricated with the HEMA resin without PEGDA-4000 is shown in Figure 4h. The wide peak at 2θ = 19.5° can be easily discerned, which implies the polymerization of HEMA resin. Meanwhile, another group of samples manufactured with the HEMA resin with the high concentration loading of PEGDA-4000 shows two peaks, one at 2θ = 5.5° and another at 2θ = 19.5° (Figure 4i), demonstrating the crystallization of PEGDA and the polymerization of HEMA, respectively. To further investigate the performance of stereolithography 3D printing with thermal assistance, a stent model was fabricated with the HEMA resin with the 40 wt% solute loading of PEGDA-4000, which is listed in Figure 4j,k. Also, Figure 4l,m shows that the models with microfeatures were 3D-printed with the HMEA resin with the 40 wt% solute loading of PEGDA-4000. The results demonstrated that the designed resin loaded with the high concentration of solute could be used for 3D printing with projection stereolithography with thermal assistance, and showed excellent performance on the fabricating structures with high resolution.

**Figure 5:**
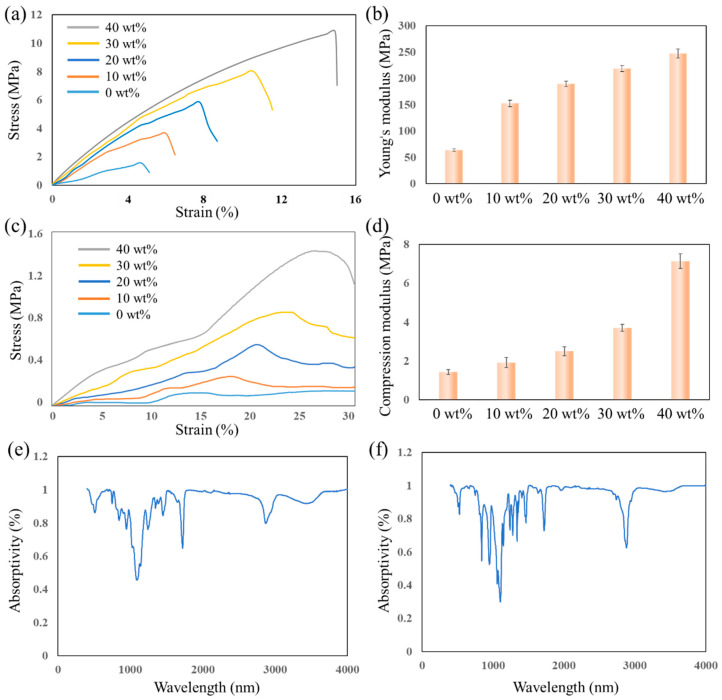
The mechanical properties test and analysis results. (**a**) The stress–strain curves of the tensile tests, (**b**) the Young’s modulus, (**c**) the stress–strain curves of the compression tests, (**d**) the compression modulus, (**e**) the infrared spectra analysis result for the HEMA resin without PEGDA-4000, and (**f**) the infrared analysis result for the HEMA resin with 40 wt% loading of PEGDA-4000.

The mechanical properties of the designed resins were studied using the tensile and compression tests. The tensile test was conducted using a rectangle sample with the dimension of 60 × 10 × 1 mm for each resin [32,33]. Figure 5a shows that the HEMA resin without PEGDA-4000 leads to the lowest stiffness as well as stretchability. As the concentration of solute PEGDA-4000 increases, both the stretchability and the maximum stress enhance significantly, which is attributed to the longer chains of PEGDA-4000 and the interfacial bond formation between HEMA and PEGDA-4000. Compared to HEMA resin without solute PEGDA-4000, HEMA resin with 40 wt% solute concentration has a three-times-higher strain. Meanwhile, compared to HEMA without solute, the maximum stress before the debonding of HEMA resin with a solute concentration of 40% has been improved by almost 10 times. Compared to the other resins, HEMA resin without PEGDA-4000 has the lowest Young’s modulus, which is 63.31 ± 2.72 MPa. As shown in Figure 5b and Table 1, the Young’s modulus of HMEA resin showed a positive effect with the concentration of solute PEGDA-4000, compared to 10 wt% HEMA resin, the Young’s modulus of 40 wt% HEMA resin has been improved from 152.62 ± 6.23 MPa to 247.14 ± 8.97 MPa, which is about 2.4 and 3.9 times that of HEMA resin without solute loading. Each error bar in Figure 5b is generated with five replicas.

For the solid part, defects are prone to generate inside the structure during 3D printing and it is hard to obtain an analyzable stress–strain curve because of the brittleness. Thus, the compression test for each resin was conducted by printing the lattice model shown in Figure 4a [34]. The compression stress–strain curves are depicted in Figure 5c, while the peak of each curve indicates the fracture in the lattice structure. Similar to the results of the tensile test, the compression strain for the HEMA resin without solute is less than 15% and the stress is about 0.1 MPa; both of them are the lowest among the designed resins. The peak stress reached about 1.4MPa and the strain raised up to 26% when the concentration of PEGDA-4000 solute in HEMA resin reached 40 wt%. Figure 5d shows the compression modulus of each resin. It is clear that the PEGDA-4000 has a positive effect on the compression modulus of the HEMA resin. As shown in Table 2, the HEMA resin without solute loading leads to a compression modulus of 1.43 ± 0.12 MPa while that modulus for the HEMA resin with 10 wt%, 20 wt%, 30 wt%, and 40 wt% solute loading of PEGDA-4000 are 1.92 ± 0.12 MPa, 2.50 ± 0.24 MPa, 3.71 ± 0.18 MPa, and 7.14 ± 0.38 MPa, respectively. In Figure 5d, each error bar is generated with five replicas.

The results of the compression and tensile tests indicated that the high loading of PEGDA-4000 is able to significantly improve the mechanical properties of HEMA resin. The infrared spectra analysis was conducted to investigate the enhancement mechanism. Figure 5e shows the analysis results for the HEMA resin without solute, in which three absorptivity peaks at 1090 nm, 1722 nm, and 2883 nm can be detected, respectively, indicating the double bonds in the polymer. However, the absorptivity peaks for the HEMA resin with 40 wt% loading of PEGDA-4000 (Figure 5f) are markedly lower than those of the HEMA resin without PEGDA-4000. This means that the double bonds decrease after the polymerization process by adding the PEGDA-4000, implying a high degree of polymerization. The infrared spectra analysis results illustrate that the PEGDA-4000 could promote the polymerization of the HEMA resin, which is consistent with the results shown in Figure 3a, and the high degree of polymerization leads to a positive effect on the mechanical properties of HEMA resin.

### 3.3. Biocompatibility Experiment

**Figure 6:**
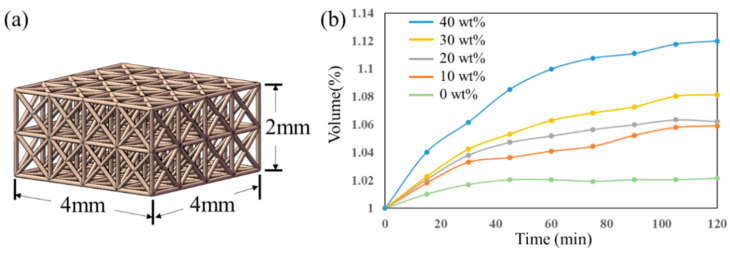
The swelling test. (**a**) The lattice model for cell proliferation, (**b**) the volume measurements for swelling.

The cell proliferation experiment was conducted to study the biocompatibility of HEMA resin loaded with the high concentration of PEGDA-4000. For comparison, lattice structures with 76% porosity are, respectively fabricated using HEMA resin without solute addition and HEMA resin with 40% solute PEGDA-4000 concentration, as shown in Figure 6a. The swelling test was executed to study the changes in volume of designed HEMA resins. Each resin was soaked in water and the volume was measured every 20 min. According to the display in Figure 6b, HEMA resin without solute loading leads to the minimal volume change while the stabilized volume increases to 102.1% of the initial volume after soaking for about 40 min. As the concentration of PEGDA-4000 increases, the swelling becomes more pronounced. The stabilized volumes rose up to 105.9%, 106.3%, 108.1%, and 111.8% of the initial volumes for the HEMA resin with PEGDA-4000 with the concentration of 10 wt%, 20 wt%, 30 wt%, and 40 wt%, respectively. The greater swelling ratio of HEMA resin loaded with the high concentration of PEGDA-4000 could contribute to the cell proliferation due to the greater surface area. Thus, the HEMA resin loaded with 40 wt% concentration of PEGDA-4000 was chosen for the cell proliferation experiment.

For the cell proliferation study, a control group and two experimental groups which were the HEMA group without PEGDA-4000 (HEMA group) and the HEMA group with 40 wt% of PEGDA-4000 (HEMA-PEGDA group) were set to verify the performance of the designed resin. For the control group, the cells were cultured without the 3D-printed scaffold. Meanwhile, for the HEMA group and the HEMA-PEGDA group, the cells were cultured with a 3D-printed HEMA scaffold and a HEMA-PEGDA scaffold, respectively.

**Figure 7:**
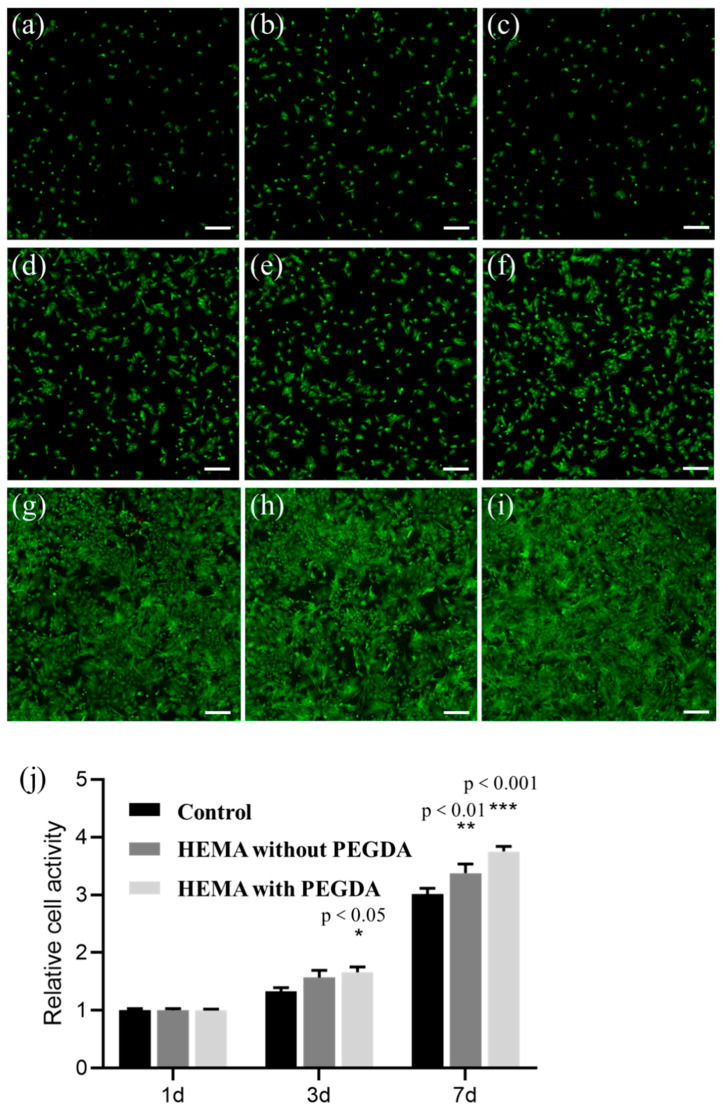
The cell proliferation experiment. (**a**–**c**) are the live/dead images of the 1-day cell culture for the control group, HEMA group, and HEMA-PEGDA group, respectively. (**d**–**f**) are the live/dead images of the 3-day cell culture for the three groups in the same order. (**g**–**i**) are the live/dead images of the 7-day cell culture. (**j**) is the relative cell activity of each group. Scale bar: 200 µm.

Figure 7 shows the fluorescence images of the cell proliferation experiment. In this test, live cells were dyed with Calcein, while dead ones were dyed with PI. When culturing cells for one day, few differences in the cell density could be detected among the three groups (Figure 7a–c). However, after culturing cells for three days, the density of the live cells was higher than that of live cells only cultured for one day for each group. And, to make a comparison between the groups after the 3-day cell culture, the density of live cells for the HEMA-PEGDA group was slightly higher than that of the other groups (Figure 7d–f). After culturing cells for seven days, the density of live cells remarkably increased for each group. Furthermore, the live cells’ density in the HEMA group was higher compared to that of the control group, while the HEMA-PEGDA group led to the highest density of live cells. The results of relative cell activity for each group can be found in Figure 7j. The relative cell activity (*R*) is assessed using Equation (3):

(3) $$R=\frac{T_{OD}}{C_{OD}}$$

where *C_OD_* is the optical density of the control group for the 1-day culture and *T_OD_* is the optical density of the target group.

The cell activity after the 1-day culture is subtly lower than that after the 3-day culture for each group, and the cell activity is the highest after the 7-day culture. For both the 3-day and 7-day cell cultures, the HEMA-PEGDA group always has a better performance and advancement in the cell activity than the others. The asterisk signs in Figure 7j represent the significance between the current group and the control group, while each significance value (*p*) was marked. The results of the cell culture prove that the HEMA resin loaded with the solute of PEGDA-4000 has an excellent performance in biocompatibility.

## 4. Conclusions

In this work, HEMA resin with the high loading of PEGDA-4000 was proved to be feasible for stereolithography 3D printing with thermal assistance using the curing depth test and microstructure fabrication at 60 °C. The compression and tensile results illustrate that the mechanical properties of HEMA resin could be remarkably improved with the high loading of PEGDA-4000. The Young’s modulus of HMEA resin is improved by 390% and the compression modulus is improved by nearly 500% with the existence of PEGDA-4000 with the concentration of 40 wt%. The cell proliferation experiments were performed to study the potential biomedical applications of the HEMA resin with the high concentration loading of PEGDA-4000. The results indicated that the HEMA resin loaded with PEGDA-4000 activates cells on a higher level compared to the HEMA resin without solute PEGDA-4000, implying that the HEMA resin loaded with a high concentration of PEGDA-4000 is an excellent candidate for biomedical research with improved mechanical performance. The results verified that the stereolithography process with thermal assistance was workable for 3D-printing the polymer loaded with high-concentration solute, through which the improved mechanical properties could be achieved. This study is expected to provide a method for the 3D printing of the polymer loaded with high concentration solute, through which the printable materials with excellent functionalities and properties are expanded.

## Figures and Tables

**Table 1:** The Young’s modulus of each resin.

	0 wt%	10 wt%	20 wt%	30 wt%	40 wt%
Young’s modulus (MPa)	63.31 ± 2.72	152.62 ± 6.23	189.75 ± 5.57	218.50 ± 5.62	247.14 ± 8.97

**Table 2:** The compression modulus of each resin.

	0 wt%	10 wt%	20 wt%	30 wt%	40 wt%
Compression modulus (MPa)	1.43 ± 0.12	1.92 ± 0.12	2.50 ± 0.24	3.71 ± 0.18	7.14 ± 0.38

## Data Availability

The data presented in this study are available on request from the corresponding author.
